# Supplementary material for: Application of concentrated growth factor in mandibular third molar extraction: A protocol for systematic review and meta-analysis
Source: PLoS One. 2024 May 2;19(5):e0302581. doi: 10.1371/journal.pone.0302581 (PMC11065272; doi:10.1371/journal.pone.0302581)
Supplement: S1 Appendix — (DOCX) [file pone.0302581.s002.docx]

Appendix 1: Search strategies

1. PubMed（<https://pubmed.ncbi.nlm.nih.gov/>）

| Search strategy for PubMed | |
| --- | --- |
| No | Search item |
| #1 | (Concentrated growth factor) OR (CGF) OR (Autologous platelet concentrate) OR (Blood platelets)) OR (Platelet concentrates) OR (Intercellular Signaling Peptides and Proteins [MeSH Terms]) OR (Blood Platelets [MeSH Terms]) OR (Blood Proteins [MeSH Terms]) OR (Fibrin [MeSH Terms]) |
| #2 | (Tooth, Impacted [MeSH Terms]) OR (Molar, Third [MeSH Terms]) OR (Molar [MeSH Terms]) OR (Third molar) OR (Impacted tooth)) OR (Wisdom tooth) |
| #3 | (Tooth Extraction [MeSH Terms]) OR (Tooth Socket [MeSH Terms]) OR (Dental extraction) OR (Tooth extraction) OR (Dental surgery) OR (Tooth Socket) |
| #4 | (randomized controlled trial) OR (randomized controlled trials as topic [MeSH Terms]) OR (randomized controlled trial [Publication Type]) |
| #5 | #2 OR #3 |
| #6 | #1 AND #4 AND #5 |

2.Web of Science(<http://www.isiknowledge.com>)

| Search strategy for Web of Science | |
| --- | --- |
| # | Search Query |
| #1 | (TS=(Concentrated growth factor) OR TS=(CGF) OR TS=(Autologous platelet concentrate) OR TS=(Blood platelets) OR TS=(Platelet concentrates) |
| #2 | (TS=(Third molar) OR TS=(Molar) OR TS=(Impacted tooth) OR TS=(Wisdom tooth) |
| #3 | (TS=(Dental extraction) OR TS=(Dental surgery) OR TS=(Tooth extraction) OR TS=(Tooth Socket) |
| #4 | #2 OR #3 |
| #5 | #1 AND #4 |

3.Embase (<https://www.embase.com>)

| Search strategy for Embase | |
| --- | --- |
| No. | Query Rseults |
| #1 | 'concentrated growth factor':ti,ab,kw OR cgf:ti,ab,kw OR 'autologous platelet concentrate':ti,ab,kw OR 'blood platelets':ti,ab,kw OR 'platelet concentrates':ti,ab,kw OR 'thrombocyte rich plasm' OR 'thrombocyte concentrate'/exp |
| #2 | 'third molar':ti,ab,kw OR molar:ti,ab,kw OR 'impacted tooth':ti,ab,kw OR 'wisdom tooth':ti,ab,kw OR 'tooth impaction'/exp OR 'third molar'/exp OR 'molar tooth'/exp OR 'mandibular third molar'/exp |
| #3 | 'dental extraction':ti,ab,kw OR 'dental surgery':ti,ab,kw OR 'tooth extraction':ti,ab,kw OR 'wisdom tooth':ti,ab,kw OR 'tooth socket':ti,ab,kw OR 'tooth extraction'/exp OR 'tooth socket'/exp |
| #4 | #2 OR #3 |
| #5 | #1 AND #4 |

4.Cochrane Library (https://www.cochranelibrary.com/)

| Search strategy for Cochrane | |
| --- | --- |
| ID | Search |
| #1 | (Concentrated growth factor):ti,ab,kw OR (CGF):ti,ab,kw OR (Autologous platelet concentrate):ti,ab,kw OR (Blood platelets):ti,ab,kw OR (Platelet concentrates):ti,ab,kw |
| #2 | (Third molar):ti,ab,kw OR (Molar):ti,ab,kw OR (Impacted tooth):ti,ab,kw OR (Wisdom tooth):ti,ab,kw |
| #3 | (Dental extraction):ti,ab,kw OR (Dental surgery):ti,ab,kw OR (Tooth extraction):ti,ab,kw OR (tooth socket):ti,ab,kw |
| #4 | #2 OR #3 |
| #5 | #1 AND #4 |

5.China National Knowledge Infrastructure (CNKI)（<https://www.cnki.net/>）

| CNKI 检索策略 | |
| --- | --- |
| No | 检索表达式 |
| #1 | （主题：浓缩生长因子）OR（主题：自体浓缩血小板）OR（主题：CGF）OR（主题：血小板） |
| #2 | （主题：阻生牙）OR（主题：下颌第三磨牙）OR（主题：阻生第三磨牙）OR（主题：智齿）OR（主题：磨牙） |
| #3 | （主题：拔牙）OR（主题：牙槽窝） |
| #4 | #2 OR #3 |
| #5 | #1 AND #4 |

6.Wanfang Database（<http://www.wanfangdata.com.cn/index.html>）

| Wanfang 检索策略（主题词扩展） | |
| --- | --- |
| No | 检索表达式 |
| #1 | 主题:(浓缩生长因子) or 主题:(CGF) or 主题:(自体浓缩血小板) or 主题:(血小板) |
| #2 | 主题:(阻生牙) or 主题:(下颌第三磨牙) or 主题:(阻生第三磨牙) or 主题:(智齿) or 主题:（磨牙） |
| #3 | 主题:(拔牙) or 主题:(牙槽窝) |
| #4 | #2 OR #3 |
| #5 | #1 AND #4 |

7.the Chinese Biological Medicine Literature Service System( <http://www.sinomed.ac.cn/>)

| CBM 检索策略 | |
| --- | --- |
| No | 检索表达式 |
| #1 | "浓缩生长因子"[全部字段] OR "CGF"[全部字段] OR "自体浓缩血小板"[全部字段] OR ("血小板"[全部字段] OR "Blood Platelets"[全部字段] OR "凝血细胞"[全部字段] OR "血小板"[主题词]) OR ("纤维蛋白"[全部字段] OR "Fibrin"[全部字段] OR "抗凝血酶Ⅰ"[全部字段] OR "纤维蛋白"[主题词]) |
| #2 | ("阻生牙"[全部字段] OR "Impacted Tooth"[全部字段] OR "牙, 阻生"[主题词]) OR "下颌第三磨牙"[全部字段] OR ("磨牙"[全部字段] OR "Molar"[全部字段] OR "磨牙"[主题词]) OR ("智齿"[全部字段] OR "第三磨牙"[全部字段] OR "Third Molar"[全部字段] OR "磨牙, 第三"[主题词]) OR "阻生第三磨牙"[全部字段] OR ("阻生牙"[全部字段] OR "Impacted Tooth"[全部字段] OR "牙, 阻生"[主题词]) |
| #3 | ("拔牙"[全部字段] OR "Tooth Extraction"[全部字段] OR "拔牙"[主题词]) OR ("牙槽窝"[全部字段] OR "Tooth Socket"[全部字段] OR "齿槽"[全部字段] OR "牙槽"[全部字段] OR "牙槽窝"[主题词]) |
| #4 | ("随机对照试验"[文献类型]) |
| #5 | #2 OR #3 |
| #6 | #1 AND #4 AND #5 |

8.VIP Database（<http://lib.cqvip.com>）

| VIP 检索策略（主题词扩展） | |
| --- | --- |
| No | 检索表达式 |
| #1 | 题名或关键词=浓缩生长因子 OR 题名或关键词=CGF OR 题名或关键词=自体浓缩血小板 OR 题名或关键词=血小板 |
| #2 | 题名或关键词=下颌第三磨牙 OR 题名或关键词=阻生牙 OR 题名或关键词=磨牙 OR 题名或关键词=阻生第三磨牙 OR 题名或关键词=智齿 |
| #3 | 题名或关键词=拔牙 OR 题名或关键词=牙槽窝 |
| #4 | #2 OR #3 |
| #5 | #1 AND #4 |

9.ProQuest Dissertations & Theses Global (<https://www.proquest.com/index>)

((Concentrated growth factor) OR (CGF) OR (Autologous platelet concentrate) OR (Blood platelets) OR (Platelet concentrates)) AND ((Third molar) OR (Molar) OR (Impacted tooth) OR (Wisdom tooth) OR (Dental extraction) OR (Dental surgery) OR (Tooth extraction) OR (tooth socket))

10.Google Scholar (<https://scholar.scholar-xm.top/>)

((Concentrated growth factor) OR (CGF) OR (Autologous platelet concentrate) OR (Blood platelets) OR (Platelet concentrates)) AND ((Third molar) OR (Molar) OR (Impacted tooth) OR (Wisdom tooth) OR (Dental extraction) OR (Dental surgery) OR (Tooth extraction) OR (tooth socket))

11.OpenGrey (<http://www.opengrey.eu>)

((Concentrated growth factor) OR (CGF) OR (Autologous platelet concentrate) OR (Blood platelets) OR (Platelet concentrates)) AND ((Third molar) OR (Molar) OR (Impacted tooth) OR (Wisdom tooth) OR (Dental extraction) OR (Dental surgery) OR (Tooth extraction) OR (tooth socket))
