# Supplementary material for: Application of concentrated growth factor in mandibular third molar extraction: A protocol for systematic review and meta-analysis
Source: PLoS One. 2024 May 2;19(5):e0302581. doi: 10.1371/journal.pone.0302581 (PMC11065272; doi:10.1371/journal.pone.0302581)
Supplement: Attachment — Submitted filename: PONE-D-24-01317_REVIEWED.pdf [file pone.0302581.s003.pdf]

PLOS ONE

Application of Concentrated Growth Factor in Mandibular Third Molar Extraction：A Protocol for Systematic Review and Meta-Analysis

--Manuscript Draft--

**Manuscript Number:**	PONE-D-24-01317
**Article Type:**	Registered Report Protocol
**Full Title:**	Application of Concentrated Growth Factor in Mandibular Third Molar Extraction：A Protocol for Systematic Review and Meta-Analysis
**Short Title:**	Concentrated Growth Factor in Mandibular Third Molar Extraction
**Corresponding Author:**	Jin Wang<br>Jinan Stamotological Hospital<br>CHINA
**Keywords:**	Concentrated Growth Factor; Mandibular Third Molar
**Abstract:**	AbstractObjective: We perform the systematic review to investigate the application of concentrated growth factor (CGF) in mandibular third molar extraction. Specifically, we aim to evaluate the effect of applying CGF on relieving postoperative complications and promoting wound healing following mandibular third molar extraction.Methods: The PubMed, Web of Science, Embase, Cochrane Library, China National Knowledge Infrastructure (CNKI), Wanfang Database, China Biology Medicine Disc (CBM), and VIP Databases will be comprehensively searched up to January 31, 2024. Randomized controlled trials (RCTs) examining the application of CGF after mandibular third molar extraction will be included. The protocol was registered in PROSPERO, and the registration ID was CRD42023463234. Two reviewers will conduct the literature search, eligible study selection, data extraction, and bias risk assessment. Data analysis will be performed with RevMan software (version 5.4).Results: The results of this study will be available in a peer-reviewed journal.Conclusion: Our study will provide scientific evidence regarding the efficacy of applying CGF in mandibular third molar extraction.
**Order of Authors:**	Hengxiao Zhang
	Jianyong Dong
	Xiaoliang Wang
	Xiaodong Sun
	Jin Wang
**Additional Information:**	
**Question**	**Response**
**Financial Disclosure**<br><br>Enter a financial disclosure statement that describes the sources of funding for the work included in this submission. Review the submission guidelines for detailed requirements. View published research articles from *PLOS ONE* for specific examples.<br><br>This statement is required for submission and **will appear in the published article** if the submission is accepted. Please make	Yes

sure it is accurate.<br><br>**Funded studies**<br>Enter a statement with the following details:<br>• Initials of the authors who received each award<br>• Grant numbers awarded to each author<br>• The full name of each funder<br>• URL of each funder website<br>• Did the sponsors or funders play any role in the study design, data collection and analysis, decision to publish, or preparation of the manuscript?<br><br>Did you receive funding for this work?	
Please add funding details.<br>as follow-up to "**Financial Disclosure**<br><br>Enter a financial disclosure statement that describes the sources of funding for the work included in this submission. Review the submission guidelines for detailed requirements. View published research articles from *PLOS ONE* for specific examples.<br><br>This statement is required for submission and **will appear in the published article** if the submission is accepted. Please make sure it is accurate.<br><br>**Funded studies**<br>Enter a statement with the following details:<br>• Initials of the authors who received each award<br>• Grant numbers awarded to each author<br>• The full name of each funder<br>• URL of each funder website<br>• Did the sponsors or funders play any role in the study design, data collection and analysis, decision to publish, or preparation of the manuscript?<br><br>Did you receive funding for this work?"	This work was supported by the Science and Technology Planning Project of Jinan Health Commission (2021-1-44).
Please select the country of your main research funder (please select carefully	CHINA - CN

as in some cases this is used in fee calculation).<br>as follow-up to "**Financial Disclosure**<br><br>Enter a financial disclosure statement that describes the sources of funding for the work included in this submission. Review the submission guidelines for detailed requirements. View published research articles from *PLOS ONE* for specific examples.<br><br>This statement is required for submission and **will appear in the published article** if the submission is accepted. Please make sure it is accurate.<br><br>**Funded studies**<br>Enter a statement with the following details:<br>• Initials of the authors who received each award<br>• Grant numbers awarded to each author<br>• The full name of each funder<br>• URL of each funder website<br>• Did the sponsors or funders play any role in the study design, data collection and analysis, decision to publish, or preparation of the manuscript?<br><br>Did you receive funding for this work?"	
**Competing Interests**<br><br>Use the instructions below to enter a competing interest statement for this submission. On behalf of all authors, disclose any competing interests that could be perceived to bias this work—acknowledging all financial support and any other relevant financial or non-financial competing interests.<br><br>This statement is required for submission and **will appear in the published article** if the submission is accepted. Please make sure it is accurate and that any funding sources listed in your Funding Information later in the submission form are also	The authors have declared that no competing interests exist.

declared in your Financial Disclosure statement.<br><br>View published research articles from *PLOS ONE* for specific examples.<br><br>**NO authors have competing interests**<br><br>Enter: *The authors have declared that no competing interests exist.*<br><br>**Authors with competing interests**<br><br>Enter competing interest details beginning with this statement:<br><br>*I have read the journal's policy and the authors of this manuscript have the following competing interests: [insert competing interests here]*<br><br>* typeset	
**Ethics Statement**<br><br>Enter an ethics statement for this submission. This statement is required if the study involved:<br><br>• Human participants<br>• Human specimens or tissue<br>• Vertebrate animals or cephalopods<br>• Vertebrate embryos or tissues<br>• Field research<br><br>Write "N/A" if the submission does not require an ethics statement.<br><br>General guidance is provided below. Consult the submission guidelines for detailed instructions. **Make sure that all information entered here is included in the Methods section of the manuscript.**	N/A

**Format for specific study types**<br><br>**Human Subject Research (involving human participants and/or tissue)**<br>• Give the name of the institutional review board or ethics committee that approved the study<br>• Include the approval number and/or a statement indicating approval of this research<br>• Indicate the form of consent obtained (written/oral) or the reason that consent was not obtained (e.g. the data were analyzed anonymously)<br><br>**Animal Research (involving vertebrate animals, embryos or tissues)**<br>• Provide the name of the Institutional Animal Care and Use Committee (IACUC) or other relevant ethics board that reviewed the study protocol, and indicate whether they approved this research or granted a formal waiver of ethical approval<br>• Include an approval number if one was obtained<br>• If the study involved *non-human primates*, add *additional details* about animal welfare and steps taken to ameliorate suffering<br>• If anesthesia, euthanasia, or any kind of animal sacrifice is part of the study, include briefly which substances and/or methods were applied<br><br>**Field Research**<br><br>Include the following details if this study involves the collection of plant, animal, or other materials from a natural setting:<br>• Field permit number<br>• Name of the institution or relevant body that granted permission	
**Data Availability**<br><br>Authors are required to make the data underlying their research findings fully available, without restriction.<br><br>If data need to be collected, modified or processed specifically for your study, or if participants need to be recruited specifically for your study, then it should	N/A - not applicable

Powered by Editorial Manager® and ProduXion Manager® from Aries Systems Corporation

occur only after your Registered Report Protocol is accepted for publication.<br><br>If the manuscript reports pilot results, the data underlying those results should be made available at the time of publication. Submission of this Registered Report Protocol implies a commitment that the data collected in the study, upon completion, will be made available with the Registered Report publication.<br><br>When legal or ethical requirements prohibit public data sharing, authors must indicate how researchers may obtain access to the data. See the PLOS Data Policy and FAQ for detailed information.<br><br>A Data Availability Statement describing where the data can be found is required at submission. Your answers to this question constitute the Data Availability Statement and **will be published in the article**, if accepted.<br><br>**Important:** Stating 'data available on request from the author' is not sufficient. If your data are only available upon request, select 'No' for the first question and explain your situation in the text box.<br><br>Do the authors confirm that all data underlying the findings of the study outlined in this Registered Report Protocol will be made fully available without restriction?	
**Describe where the data may be found in full sentences. If you are copying our sample text, replace any instances of XXX with the appropriate details.**<br><br>• If **no pilot data are reported in the manuscript**, please enter the following: *All relevant data from this study will be made available upon study completion.*	No datasets were generated or analysed during the current study.

Powered by Editorial Manager® and ProduXion Manager® from Aries Systems Corporation

• If the manuscript reports pilot data **held in a public repository**, include URLs, accession numbers or DOIs. If this information will only be available after acceptance, indicate this by ticking the box below. For example: *All XXX files are available from the XXX database (accession number(s) XXX, XXX.).*<br>• If the manuscript reports pilot data contained **within the manuscript and/or Supporting Information files**, enter the following:<br>*All relevant data are within the manuscript and its Supporting Information files.*<br>• If neither of these applies but you are able to provide **details of access elsewhere**, with or without limitations, please do so. For example:<br><br>*Data cannot be shared publicly because of [XXX]. Data are available from the XXX Institutional Data Access / Ethics Committee (contact via XXX) for researchers who meet the criteria for access to confidential data.*<br><br>*The data underlying the results presented in the study are available from (include the name of the third party and contact information or URL).*<br>• This text is appropriate if the data are owned by a third party and authors do not have permission to share the data.<br><br>* typeset	
Additional data availability information:	Tick here if your circumstances are not covered by the questions above and you need the journal's help to make your data available.

Application of Concentrated Growth Factor in Mandibular Third Molar Extraction：A Protocol for Systematic Review and Meta-Analysis

Hengxiao Zhang[1]¶, Jianyong Dong[1]¶, Xiaoliang Wang[1], Xiaodong Sun[1], Jin Wang[1*]

[1] Gaoxin Branch, Jinan Stomatological Hospital, Jinan 250000, Shandong Province, China

¶These authors contributed equally to this work.

*634833675@qq.com

Funding: This work was supported by the Science and Technology Planning Project of Jinan Health Commission (2021-1-44).

This is a systematic review which does not require ethical approval.

All authors have read and approved the final manuscript.

Abstract

Objective: We perform the systematic review to investigate the application of concentrated growth factor (CGF) in mandibular third molar extraction. Specifically, we aim to evaluate the effect of applying CGF on relieving postoperative complications and promoting wound healing following mandibular third molar extraction.

Methods: The PubMed, Web of Science, Embase, Cochrane Library, China National Knowledge Infrastructure (CNKI), Wanfang Database, China Biology

Medicine Disc (CBM), and VIP Databases will be comprehensively searched up to January 31, 2024. Randomized controlled trials (RCTs) examining the application of CGF after mandibular third molar extraction will be included. The protocol was registered in PROSPERO, and the registration ID was CRD42023463234. Two reviewers will conduct the literature search, eligible study selection, data extraction, and bias risk assessment. Data analysis will be performed with RevMan software (version 5.4).

Results: The results of this study will be available in a peer-reviewed journal.

Conclusion: Our study will provide scientific evidence regarding the efficacy of applying CGF in mandibular third molar extraction.

Introduction

The extraction of the mandibular third molar is a common oral and maxillofacial procedure. Following the procedure, various types of postoperative complications occur, including pain, swelling, and trismus[1]. Various interventions have been applied to reduce these complications, such as corticosteroid therapy, antibiotic regimens, piezosurgery, laser therapy, cryotherapy, and bioactive surgical additives[2]. In addition to short-term postoperative adverse events, the extraction of mandibular third molars lead to long-term damage to the periodontal tissue around second molars, which cannot be ignored[3]. The extraction of an impacted third molar can result in a residual alveolar defect, the loss of attachment, and periodontal pocket

formation at distal sites of second molars[4]. However, challenges remain with respect to relieving patient discomfort, accelerating wound healing and promoting tissue regeneration.

Blood can be used to extract concentrated growth factor (CGF), a third-generation platelet concentrate[5]. CGF has many advantages compared with first- and second-generation platelet concentrates, i.e., platelet-rich plasma (PRP) and platelet-rich fibrin (PRF)[6]. The growth factors and $CD34^{+}$ stem cells in CGF increase its ability to promote bone formation and tissue healing. CGF has a harder, larger and denser fibrin structure and serves as a scaffold at the application site, thus promoting tissue regeneration[7]. CGF can promote wound healing, improve the regeneration of bone and soft tissue, and decrease inflammation. It has been widely used in oral and maxillofacial procedures, such as oral implants, maxillary sinus floor elevation, periodontal tissue regeneration, alveolar ridge preservation and tooth extraction[8-10]. CGF has been demonstrated to be beneficial for decreasing the incidence of postoperative complications[11], healing soft tissue[12] and promoting ridge preservation[13] after third molar surgery. New studies about the promising efficacy of CGF in third molar extraction are constantly emerging. However, there are some inconclusive results. Some studies have indicated that CGF has no additional beneficial effects on pain, swelling, or trismus after surgery[14]. Another study revealed that CGF had no positive effects on the distal periodontal depth or bone

regeneration of the second molar[15]. However, these previous studies had small sample sizes, insufficient analysis factors, methodological differences and shorter follow-up periods, thus preventing us from drawing firm conclusions.

To our knowledge, given the small number of relevant randomized controlled trials (RCTs), no systematic review has been conducted on this specific topic. There is limited scientific evidence supporting the widespread clinical application of CGF in mandibular third molar extraction. Because more articles have recently been published on this subject, we conduct this meta-analysis. The objectives of the current review are as follows: 1) to determine whether CGF relieves postoperative clinical outcomes (pain, swelling, and trismus) in the short term; 2) to evaluate whether CGF promotes the healing of soft tissue; and 3) to determine whether CGF promotes periodontal regeneration (soft tissue and bone healing) in the distal area of the adjacent second molar following mandibular third molar surgery.

Methods

This systematic review will be conducted in accordance with the PRISMA guidelines[16]. The PRISMA-P checklist is included in S1 Table. The protocol has been registered in PROSPERO (CRD42023463234). The PICOS strategy was used to construct the following research parameters: (1) population (P): patients who underwent mandibular third molar extraction; (2) intervention (I) and comparison (C): in the intervention group, CGF was placed into the tooth socket, while no treatment

was used in the control group; (3) outcomes (O): the primary outcome and secondary outcome are described below; and (4) study design (S): RCTs including human subjects. The research question is as follows: Does the application of CGF relieve postoperative clinical outcomes, improve the healing of soft tissue and promote regeneration of periodontia after mandibular third molar extraction?

Information sources and search strategy

Two independent reviewers (Hengxiao Zhang and Jianyong Dong) will search the PubMed, Web of Science, Embase, Cochrane Library, China National Knowledge Infrastructure (CNKI), Wanfang Database, China Biology Medicine Disc (CBM), and VIP Databases to identify potentially relevant articles. We will resolve disagreements by consulting a third reviewer (Jin Wang). The search strategy will be developed by a combination of medical subject terms (MeSH) and free text words. The free text words will include "concentrated growth factor" or "CGF", and "third molar". RCTs that included human subjects and were published up to January 31, 2024 will be considered. The search strategy for PubMed is presented in Table 1. This strategy will be adapted for the other database searches.

Study selection

Two independent reviewers (Hengxiao Zhang and Jianyong Dong) will screen the studies. We will import all of the retrieved studies into Endnote software (version

X9), and duplicate articles will be automatically excluded. After screening the title and abstract, we will obtain the full text of the relevant research. The reviewers will assess whether each article meets the inclusion criteria to determine its eligibility. We will also manually search the reference lists of the included articles to identify additional relevant studies. If there is any disagreement, it will be decided by the third reviewer (Jin Wang). The main reasons for the exclusion of articles and the research selection process will be documented. The PRISMA flow chart is shown in Figure 1.

Inclusion and exclusion criteria

The inclusion criteria will be as follows: 1) randomized clinical trials, 2) studies evaluating the use of CGF in mandibular third molar extraction, 3) studies examining pain, swelling, trismus, or soft tissue healing as primary or secondary outcomes, and 4) studies mainly published in Chinese or English. The relevant exclusion criteria will be as follows: 1) retrospective studies, 2) studies using CGF along with other elements, and 3) studies for which relevant data were not reported.

Primary outcome and secondary outcome

The removal of mandibular third molars is often accompanied by unavoidable adverse effects, such as pain, swelling, and trismus. The primary outcome will be postoperative pain. The secondary outcomes will include facial swelling, trismus, soft tissue healing, bone height and periodontal parameters (i.e., probing depth, gingival

recession and clinical attachment level).

Data extraction

A previously prepared data extraction form will be used. The details will be extracted: authors, year, study design, participant age, male-to-female ratio, number of patients, interventions, flap design, bone removal, operation time, primary and secondary outcomes, specific measurement methods, and duration of follow-up. Two reviewers (Hengxiao Zhang and Jianyong Dong) will extract the data in accordance with the inclusion criteria. In cases of disagreement, the third author will resolve the inconsistencies. If important data are missing, we will contact the corresponding authors.

Risk of bias assessment

Two independent reviewers will assess the risk of bias using the Cochrane Collaboration risk-of-bias tool for randomized controlled trials [17]. The following domains will be evaluated: random sequence generation, allocation concealment, blinding of participants and personnel, blinding of outcome assessment, incomplete data, selective reporting, and risk of other potential bias. Each study will be graded as unclear, low, or high risk.

Assessment of heterogeneity and data synthesis

All meta-analyses will be conducted using RevMan software (version 5.4). Calculation of weighted mean difference (WMD) and 95% confidence intervals (CIs) will be necessary for continuous data. In case of data are presented as medians and quartiles, we will convert them to means and standard deviations (SDs)[18]. For dichotomous variables, we will calculate risk ratios (RRs) or odds ratios (ORs) along with 95% CIs. Cochrane's Q test and the I^2 statistic will be used to assess heterogeneity of the studies[19]. If significant heterogeneity ($P < 0.05$ and $I^2 > 50\%$) is observed, the random effects model will be adopted, which can provide a more conservative estimate of the difference. In the cases of low heterogeneity, we will employ the fixed effect model to assure the statistical efficiency. To account for methodological heterogeneity in the measurements, a descriptive synthesis will be performed if variables could not be included in the meta-analysis.

Subgroup analysis

If significant heterogeneity is observed, we will conduct subgroup analysis based on sex, position of the mandibular third molar, flap design, bone removal and duration of surgery.

Sensitivity analysis

We will assess the stability and reliability of the results through sensitivity analysis. We will eliminate one study at a time to determine if the study account for significant

heterogeneity.

Publication bias

If we include 10 or more articles, we will conduct publication bias by Begg's and Egger's tests[20]. The results will be presented by a funnel plot. An asymmetry in the funnel plot will be considered to indicate potential publication bias.

Grading the quality of evidence

We will use the Grading of Recommendations Assessment, Development and Evaluation (GRADE) framework to assess the quality of evidence[21]. The quality of evidence will be categorized into 4 levels: very low, low, moderate, or high.

Discussion

This systematic review and meta-analysis of published RCTs will be performed to evaluate the application of CGF for mandibular third molar extraction. This review could help clinicians better understand the potential advantages of CGF in third molar extraction. Many studies have proven the effectiveness of CGF in the extraction of mandibular third molar; however, there is no consensus. Most related studies have focused on short-term improvements in postoperative symptoms[12]. However, few relevant studies have explored the roles of CGF in promoting of periodontal tissue regeneration of third molars and adjacent second molars[13]. Our study will review

the currently available evidence regarding the use of CGF in mandibular third molar extraction. This review has several limitations. Differences in surgical protocols and the outcome measurements may lead to bias in the results. This review will only include English and Chinese studies, which may decrease the generalizability of the review. Our review will draw comprehensive quantitative and qualitative conclusions based on the currently available evidence. The results of the study will provide evidence-based medical guidance for clinicians regarding the use of CGF. Furthermore, these findings will illustrate the weaknesses of current studies and can inform future research.

Supporting information

S1 Checklist. PRISMA-P (Preferred Reporting Items for Systematic review and Meta-Analysis Protocols) 2015 checklist. (DOC)

Authors' Contributions

Conceptualization: Hengxiao Zhang, Jianyong Dong, Jin Wang.

Data curation: Hengxiao Zhang, Jianyong Dong, Xiaoliang Wang, Xiaodong Sun.

Formal analysis: Hengxiao Zhang, Jianyong Dong, Xiaoliang Wang, Xiaodong Sun.

Funding acquisition: Jianyong Dong.

Investigation: Hengxiao Zhang, Jin Wang.

Methodology: Hengxiao Zhang, Jianyong Dong.

Project administration: Hengxiao Zhang, Jin Wang.

Supervision: Jin Wang.

Validation: Hengxiao Zhang, Jianyong Dong, Jin Wang.

Writing – original draft: Hengxiao Zhang, Jianyong Dong.

Writing – review & editing: Jin Wang.

Declaration of Conflicting Interests

The authors declared no potential conflicts of interest with respect to the research, authorship, and/or publication of this article.

Ethics and Dissemination

Ethical approval is not applicable since this is a protocol for systematic review and meta-analysis that does not include actual patients or require data privacy.

Funding

This work was supported by the Science and Technology Planning Project of Jinan Health Commission (2021-1-44).

References

[1] O.D. Osunde, R.A. Adebola, U.K. Omeje, Management of inflammatory complications in third molar surgery: a review of the literature, Afr Health Sci 11(3) (2011) 530-7.

[2] H. Cho, A.J. Lynham, E. Hsu, Postoperative interventions to reduce inflammatory complications after third molar surgery: review of the current evidence, Aust Dent J 62(4) (2017) 412-419.
[3] Z.B. Li, H.L. Qu, L.N. Zhou, B.M. Tian, F.M. Chen, Influence of Non-Impacted Third Molars on Pathologies of Adjacent Second Molars: A Retrospective Study, J Periodontol 88(5) (2017) 450-456.
[4] Y.Y. Leung, A.W.K. Yeung, I.N. Ismail, N.S.M. Wong, Bone regeneration at the distal aspect of the adjacent second molar after lower third molar coronectomy: a long-term analysis, Int J Oral Maxillofac Surg 49(10) (2020) 1360-1366.
[5] L.F. Rodella, G. Favero, R. Boninsegna, B. Buffoli, M. Labanca, G. Scarì, L. Sacco, T. Batani, R. Rezzani, Growth factors, CD34 positive cells, and fibrin network analysis in concentrated growth factors fraction, Microsc Res Tech 74(8) (2011) 772-7.
[6] E. Marchetti, L. Mancini, S. Bernardi, S. Bianchi, L. Cristiano, D. Torge, G. Marzo, G. Macchiarelli, Evaluation of Different Autologous Platelet Concentrate Biomaterials: Morphological and Biological Comparisons and Considerations, Materials (Basel) 13(10) (2020).
[7] F. Ma, Y. Lin, F. Sun, X. Jiang, T. Wei, The impact of autologous concentrated growth factors on the alveolar ridge preservation after posterior tooth extraction: A prospective, randomized controlled clinical trial, Clin Implant Dent Relat Res 23(4) (2021) 579-592.
[8] L. Chen, J. Cheng, Y. Cai, J. Zhang, X. Yin, Q. Luan, Efficacy of concentrated growth factor (CGF) in the surgical treatment of oral diseases: a systematic review and meta-

analysis, BMC Oral Health 23(1) (2023) 712.

[9] M. Ghasemirad, M.T. Chitsazi, M. Faramarzi, L. Roshangar, A. Babaloo, R. Chitsazha, Histological examination of the effect of concentrated growth factor (CGF) on healing outcomes after maxillary sinus floor augmentation surgery, J Med Life 16(2) (2023) 267-276.

[10] S.K. Akcan, B. Ünsal, Gingival recession treatment with concentrated growth factor membrane: a comparative clinical trial, J Appl Oral Sci 28 (2020) e20190236.

[11] B. Özveri Koyuncu, G. Işık, M. Özden Yüce, S. Günbay, T. Günbay, Effect of concentrated growth factor (CGF) on short-term clinical outcomes after partially impacted mandibular third molar surgery: A split-mouth randomized clinical study, J Stomatol Oral Maxillofac Surg 121(2) (2020) 118-123.

[12] S.A. Elayah, X. Liang, K.A. Sakran, L. Xie, H. Younis, A.E. Alajami, J. Tu, S. Na, Effect of concentrated growth factor (CGF) on postoperative sequel of completely impacted lower third molar extraction: a randomized controlled clinical study, BMC Oral Health 22(1) (2022) 368.

[13] S.A. Elayah, H. Younis, H. Cui, X. Liang, K.A. Sakran, B. Alkadasi, E.A. Al-Moraissi, M. Albadani, W. Al-Okad, J. Tu, S. Na, Alveolar ridge preservation in post-extraction sockets using concentrated growth factors: a split-mouth, randomized, controlled clinical trial, Front Endocrinol (Lausanne) 14 (2023) 1163696.

[14] D. Torul, M.M. Omezli, K. Kahveci, Evaluation of the effects of concentrated growth factors or advanced platelet rich-fibrin on postoperative pain, edema, and trismus following lower third molar removal: A randomized controlled clinical trial, J Stomatol Oral Maxillofac Surg 121(6) (2020) 646-651.

[15] D. Fang, D. Li, C. Li, W. Yang, F. Xiao, Z. Long, Efficacy and Safety of Concentrated Growth Factor Fibrin on the Extraction of Mandibular Third Molars: A Prospective, Randomized, Double-Blind Controlled Clinical Study, J Oral Maxillofac Surg 80(4) (2022) 700-708.

[16] D. Moher, A. Liberati, J. Tetzlaff, D.G. Altman, P.G. The, Preferred Reporting Items for Systematic Reviews and Meta-Analyses: The PRISMA Statement, PLOS Medicine 6(7) (2009) e1000097.

[17] J.P. Higgins, D.G. Altman, P.C. Gøtzsche, P. Jüni, D. Moher, A.D. Oxman, J. Savovic, K.F. Schulz, L. Weeks, J.A. Sterne, The Cochrane Collaboration's tool for assessing risk of bias in randomised trials, Bmj 343 (2011) d5928.

[18] D. Luo, X. Wan, J. Liu, T. Tong, Optimally estimating the sample mean from the sample size, median, mid-range, and/or mid-quartile range, Stat Methods Med Res 27(6) (2018) 1785-1805.

[19] J. Lau, J.P. Ioannidis, C.H. Schmid, Quantitative synthesis in systematic reviews, Ann Intern Med 127(9) (1997) 820-6.

[20] M. Egger, G. Davey Smith, M. Schneider, C. Minder, Bias in meta-analysis detected by a simple, graphical test, Bmj 315(7109) (1997) 629-34.

[21] G. Guyatt, A.D. Oxman, E.A. Akl, R. Kunz, G. Vist, J. Brozek, S. Norris, Y. Falck-Ytter, P. Glasziou, H. DeBeer, R. Jaeschke, D. Rind, J. Meerpohl, P. Dahm, H.J. Schünemann, GRADE guidelines: 1. Introduction-GRADE evidence profiles and summary of findings tables, J Clin Epidemiol 64(4) (2011) 383-94.

Figure 1

Click here to access/download;Figure;PRISMA 2020 flow diagram 20231221-01.tif

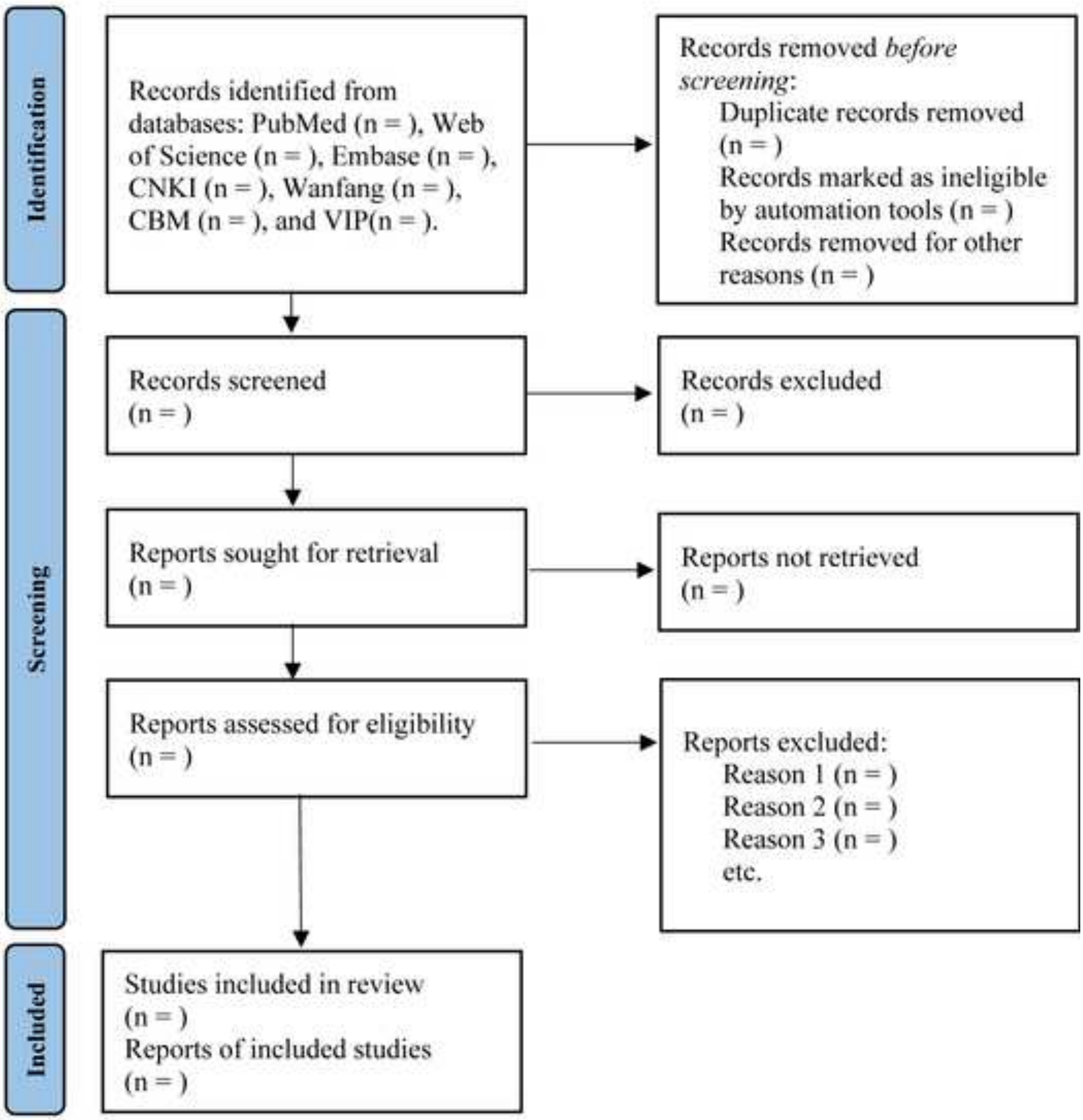

Figure 1. Flow chart and descriptions of study selection.

Click here to access/download;Figure;Pubmed.docx

Table 1

Details of the search strategy for PubMed

No	Search item
#1	("concentrate"[All Fields] OR "concentrated"[All Fields] OR "concentrates"[All Fields] OR "concentrating"[All Fields] OR "concentration"[All Fields] OR "concentrations"[All Fields]) AND ("intercellular signaling peptides and proteins"[MeSH Terms] OR ("intercellular"[All Fields] AND "signaling"[All Fields] AND "peptides"[All Fields] AND "proteins"[All Fields]) OR "intercellular signaling peptides and proteins"[All Fields] OR ("growth"[All Fields] AND "factor"[All Fields]) OR "growth factor"[All Fields])
#2	"CGF"[All Fields]
#3	("blood platelets"[MeSH Terms] OR ("blood"[All Fields] AND "platelets"[All Fields]) OR "blood platelets"[All Fields] OR "platelet"[All Fields] OR "platelets"[All Fields] OR "platelet s"[All Fields] OR "plateletes"[All Fields]) AND ("concentrate"[All Fields] OR "concentrated"[All Fields] OR "concentrates"[All Fields] OR "concentrating"[All Fields] OR "concentration"[All Fields] OR "concentrations"[All Fields])
#4	"molar, third"[MeSH Terms] OR ("molar"[All Fields] AND "third"[All Fields]) OR "third molar"[All Fields] OR ("third"[All Fields] AND "molar"[All Fields])
#5	"molar, third"[MeSH Terms] OR ("molar"[All Fields] AND "third"[All Fields]) OR "third molar"[All Fields] OR ("wisdom"[All Fields] AND "tooth"[All Fields]) OR "wisdom tooth"[All Fields]
#6	#1 OR #2 OR #3
#7	#4 OR #5
#8	"randomized controlled trial"[Publication Type] OR "randomized controlled trials as topic"[MeSH Terms] OR "randomized controlled trials"[All Fields] OR "randomised controlled trials"[All Fields]
#9	#6 AND #7 AND #8

Click here to access/download
Supporting Information
PRISMA-P 2015 checklist 20240115.doc
